# The Penrose Effect and its acceleration by the war on drugs: a crisis of untranslated neuroscience and untreated addiction and mental illness

**DOI:** 10.1038/s41398-019-0661-9

**Published:** 2019-11-28

**Authors:** Gregory G. Grecco, R. Andrew Chambers

**Affiliations:** 1 Medical Scientist Training Program, Indiana University of School of Medicine, Indianapolis, IN USA; 2 Stark Neurosciences Research Institute, Indiana University School of Medicine, Indianapolis, IN USA; 3 Department of Psychiatry, Indiana University School of Medicine, Indianapolis, IN USA; 4 Laboratory for Translational Neuroscience of Dual Diagnosis & Development, IU Neuroscience Research Center, Indianapolis, IN USA

**Keywords:** Addiction, Neuroscience

## Abstract

In 1939, British psychiatrist Lionel Penrose described an inverse relationship between mental health treatment infrastructure and criminal incarcerations. This relationship, later termed the ‘Penrose Effect’, has proven remarkably predictive of modern trends which have manifested as reciprocal components, referred to as ‘deinstitutionalization’ and ‘mass incarceration’. In this review, we consider how a third dynamic—the criminalization of addiction via the ‘War on Drugs’, although unanticipated by Penrose, has likely amplified the Penrose Effect over the last 30 years, with devastating social, economic, and healthcare consequences. We discuss how synergy been the Penrose Effect and the War on Drugs has been mediated by, and reflects, a fundamental neurobiological connection between the brain diseases of mental illness and addiction. This neuroscience of dual diagnosis, also not anticipated by Penrose, is still not being adequately translated into improving clinical training, practice, or research, to treat patients across the mental illness-addictions comorbidity spectrum. This failure in translation, and the ongoing fragmentation and collapse of behavioral healthcare, has worsened the epidemic of untreated mental illness and addictions, while driving unsustainable government investment into mass incarceration and high-cost medical care that profits too exclusively on injuries and multi-organ diseases resulting from untreated addictions. Reversing the fragmentation and decline of behavioral healthcare with decisive action to co-integrate mental health and addiction training, care, and research—may be key to ending criminalization of mental illness and addiction, and refocusing the healthcare system on keeping the population healthy at the lowest possible cost.

## Introduction

Mental illness, addictions, and their comorbid variants (termed ‘dual diagnoses’) are poorly addressed by the U.S. healthcare system due to relatively low insurance reimbursement, declining treatment infrastructure, and insufficient professional training—allowing these brain diseases to produce an increasingly large burden of secondary medical, economic, and social consequences^1–3^. For Americans under 50, suicide and overdoses involving addictive drugs are now both ranked in the top four leading proximal causes of death^4,5^. As root cause pathologies that eventually generate many other secondary organ injuries, cardiovascular diseases, cancers and infections, undertreated addictions collectively represent the largest public health threat and cause of early death in the U.S^6–8^.

Toward unraveling the origins and complexities of this healthcare crisis, at the core of which persists an increasingly deficient behavioral healthcare system, this review explores the synergistic effects of three long-standing, interactive phenomena^1^: the ‘Penrose Effect’^2^; the ‘War on Drugs’^3^; and a failure in translation of the Neuroscience of Dual Diagnosis (i.e. the science that describes the biological connections between addictions and mental illness). Explaining these trends and their interactive dynamics, requires a weaving together of historical, sociological, and neuroscientific perspectives, that may inform a strategy for re-building behavioral healthcare as a core rather than marginal domain of the healthcare system. This rebuilding, involving a more complete, mainstream integration of addiction and mental health services, professional training and research, may be crucial for decisively reducing the twin challenges of mass incarceration and the modern behavioral healthcare crisis.

## The Penrose Effect

**Fig. 1:**
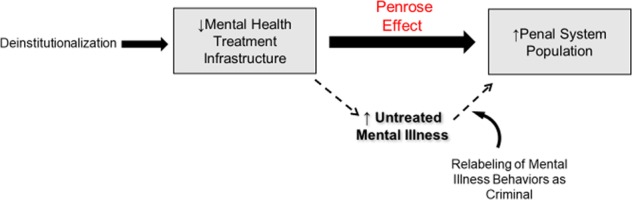
The Penrose Effect describes the inverse relationship between mental health treatment infrastructure and prison populations. Dotted arrows indicate one pathway underlying the Penrose Effect in which removal of mental health treatment resources leads to the criminalization of untreated mental illness resulting in increased numbers of individuals with mental illness in correctional facilities.

In 1939, Lionel Penrose published his seminal theory that in industrialized nations, a decline of mental health treatment infrastructure is linked with reciprocal increases in incarcerations^9^ (Fig. 1). His analysis included cross-sectional data from 18 European countries, showing an inverse relationship between national volumes of psychiatric beds and numbers of prisoners and crime measures^9^. This theory, subsequently termed the “Penrose Effect”^10^, “Penrose Hypothesis”^11^, or “Penrose Law”^12^, has been increasingly referred to in modern studies examining the unintended consequences of deinstitutionalization^13^.

Beginning in the late 1960s, large-scale deinstitutionalization commenced with depopulating and closure of long-term inpatient psychiatric hospitals with plans to expand outpatient community mental health services^14,15^. The movement was driven by several agendas: to cut costs, to improve civil rights and social integration for patients, and (in what has proven to be an over-optimistic appraisal of the therapeutic power of pharmaceuticals) to take advantage of a growing repertoire of psychiatric medications to promote healing aside from psychotherapies and environmental settings^14–16^.

Problems with long-term psychiatric institutions were widely publicized following the Rosenhan Experiment, which highlighted the powerlessness, dehumanization, and isolation of patients^17^. Stanford psychologist David Rosenhan and seven other “pseudopatients” feigned auditory hallucinations to gain admissions into psychiatric wards, with plans to stop reporting symptoms immediately after entering. The pseudopatients were subsequently administered numerous psychotropic medications and were stuck for an average of 19 days in these institutions. Rosenhan concluded that community-based health approaches should be pursued due to the counter-therapeutic environment of institutionalization^17^. However, there was little evidence at the time to suggest community-based health services would be built to compensate for declining inpatient psychiatric services, or that they would be less expensive^14,15,18^. Unfortunately, deinstitutionalization was poorly organized and conducted without adequate build-up of supportive housing, social services, or outpatient-community mental health infrastructure^19,20^. Thus, deinstitutionalization has not only created unprecedented waves of homelessness, but it has, consistent with the Penrose Effect, forced the criminal justice system to assume the role of mental healthcare, as untreated, unsheltered individuals were relabeled as “criminals”^21–24^.

As deinstitutionalization has progressed, evidence for the Penrose Effect has accumulated internationally including Argentina, Bolivia, Brazil, Chile, Paraguay, and Uruguay^20^, Norway^25^, Hungary^26^, Ireland^27^, Finland^28^, England, Spain, Sweden, Germany, the Netherlands^29,30^, Australia^31^, and the U.S.^32–35^. Mundt et al. provides a notable demonstration of a direct association between deinstitutionalization and increasing prison populations in six of the most populous countries in South America from 1991 to 2012.^20^ Even after controlling for variance in macroeconomic changes across these countries, the numbers of psychiatric hospital beds had greatly reduced where and when prison populations increased^20^.

Although both cross-sectional and longitudinal data have been replicated in support of the Penrose Effect, controversy remains about what mechanisms are driving it, and/or whether it is an artifact of other phenomena, such as changes in income inequality, unemployment, macro-economies, social welfare programs, and/or changes in national healthcare systems^11,13,14,19,36^. Nevertheless, the existence of multiple forces contributing to the inverse relationship between mental health treatment infrastructure and prison populations does not preclude the existence of the Penrose Effect, nor its consequences; it only suggests the Penrose Effect is a complex phenomenon.

One dynamic that may underpin the Penrose Effect is increased labeling and categorization of behaviors, resulting from untreated and unsheltered mental illness, as minor forms of criminal activity, which would lead to greater incarceration rates for mentally ill people. The average number of state psychiatric hospital beds (U.S.) has dropped from 339 per 100,000 in 1955 to ~14 per 100,000 in 2010 leaving 3.2 million individuals with serious mental illness to reside in the community^22,37,38^. Mental illness rates and severity in U.S. prisons rose steeply during this time. In 1870, <1% of prisoners had a serious mental illness, yet today, that number is around 20% by conservative estimates^22,39^. By 2012, there were nearly 10 times the number of persons with a mental illness in prisons than in psychiatric hospitals^39^. In nearly every U.S. state, a correctional facility holds more inmates with a mental illness than the largest state psychiatric hospital^39^.

Although antisocial traits and diagnoses are present within mentally ill populations, this form of mental illness only represents a small fraction of patients, and does not represent the diversity of many other serious mental disorders for which incarceration has replaced access to appropriate treatment services and professionals^22,37^. Without easy access to expert psychiatric treatment and supportive, supervised housing, individuals with mental illness navigate a society that has placed the criminal justice system in front of, or between them and treatment^40,41^. Consequently, a substantial population of mentally ill now reside in correctional facilities^40^.

Remarkably, this state of affairs is not new. Despite advances in psychiatric neuroscience, and the destigmatizing effects this science was hoped to bring, the U.S. seems to have regressed significantly to a more primitive approach that was the norm before the U.S. Civil War, in which punishment was the first line approach for many patients. In the 1840s, Dorothea Dix and other advocates began to challenge state legislatures regarding the inhumane treatment of mentally ill people in prisons; this activism later spearheaded the building of a national mental healthcare infrastructure and hospital system that aimed to put treatment in front of punishment^42,43^.

## The War on Drugs

**Fig. 2:**
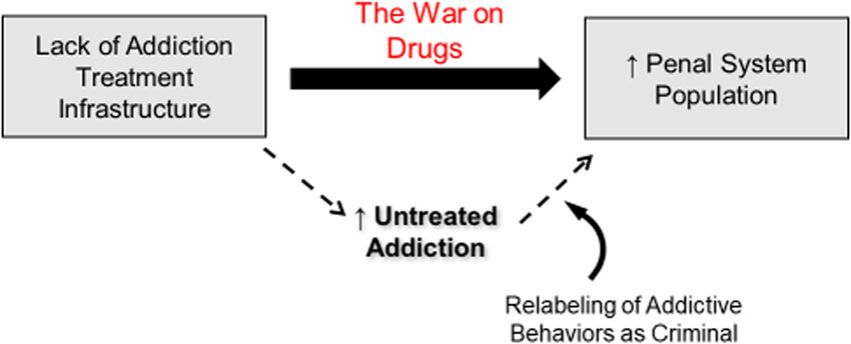
The War on Drugs describes a variety of legal policies that attempt to solve addiction via criminal-justice and punitive actions rather than biomedical-treatment approaches. The War on Drugs has been shown to be a key driver in penal system expansion. The dotted arrows indicate a pathway by which the criminalization of behavior related to untreated addiction has led to increased prison populations resulting from drug-related offenses.

The policy of addressing compulsive drug use (i.e. addiction) as a crime in the modern era is commonly referred to as “the War on Drugs” (Fig. 2). The War on Drugs massively increased drug enforcement spending, the scope of federal drug task forces^44,45^, and drug-related arrests^46,47^. Although Prohibition in the 1920s can be viewed as a failed, alcohol-specific prequel to the War on Drugs^48,49^, the modern War on non-alcohol intoxicants was formally launched by President Nixon in the early 1970s^44^. Since this time, the U.S. government, through bipartisan decisions, has expended more than $1 trillion on the War on Drugs at roughly $51 billion annually, which is about 50 times the annual research budget on addiction supported by the National Institute on Drug Abuse^46^. Subsequent “tough on crime” policies produced substantial increases in state, local, and federal police funding between 1992 and 2008^44,45^. This growth in the size and power of the criminal justice system, has been accompanied by growing concerns about its militarization, suppression of civil rights, and conduct operating outside of constitutional law^45,50–52^. For example, the civil forfeiture of assets in drug-related cases has permitted law enforcement agencies to seize around $7 billion in assets between 1985 and 1999 without due process rights^50,52^.

The War on Drugs has been enacted as a criminal-legal approach to addiction in sharp contrast to biomedical approaches, fundraising, and destigmatizing efforts that have also been labeled as “Wars” on other diseases. For example, the “War on Cancer” was also declared in the 1970s under the Nixon administration but as an entirely biomedical approach to find cures^53,54^. Few would argue that the War on Cancer has been “won”; however, significant strides in our understanding of cancer biology have led to dozens of innovations in early detection and cancer therapies^53,54^. Conversely, the War on Drugs has not encouraged progress in treatment delivery or development and has failed to reduce drug supply, drug-related crime, or addiction disease rates and death^46,52,55–57^. In fact, the War on Drugs has been shown to promote barriers to treatment and to increase drug-related violence globally^46,47,55^. Likewise, viewing addiction as a criminal behavior has resulted in increased stigmatization, facilitated discriminatory racial policies, and is linked with increased overdoses and the transmission rates of HIV, hepatitis, and tuberculosis^46,47,56–58^.

Another widely recognized consequence of the War on Drugs is the increased incarceration rate^46,47^. The number of arrests for drug possession between 1982 and 2007 rose from ~500,000 to 1.5 million, with arrests for all other offenses (excluding assaults) declining during the same timeframe^44,45^. This incarceration rate has allowed the U.S. to become the world’s leader in per capita incarcerations; although making up just 5% of the world’s population, it contains 25% of the world’s entire prison population^44,45,59,60^.

Comparable to how labeling mental illness-related behavior as a crime may drive the Penrose Effect, the War on Drugs has perpetuated beliefs, held by the public, including members and stakeholders of the healthcare industry, that addiction is a moral and criminal problem, and not a biomedical problem. Although the U.S. biomedical research system has accumulated overwhelming scientific proof that addiction is a highly prevalent brain disease and a leading root cause of premature illness, injury and death, the healthcare system ironically remains extremely ill-equipped to adequately diagnose and treat it^6–8,61,62^. Beyond this incapacity, the lack of professional awareness and training about addictive diseases among physicians has allowed the U.S. healthcare system to directly contribute to the single largest outbreak of serious addiction (and related illness and death) in U.S. history—through the iatrogenic opioid epidemic^63^. Although the opioid epidemic may have finally increased awareness of addiction as a treatable brain disease, the building of widely accessible treatment services and physician expertise needed to address the epidemic has proven elusive^64,65^. The road to parity of quality care for mental illness and addictions (that is on par with treatment of medical disease of body organs) remains largely blocked by profound shortages of behavioral health professionals and infrastructure, as well as entrenched stigma and financial incentives that benefit from keeping addiction framed as a criminal-legal issue rather than a biomedical problem^40,61,66^.

## The addiction–mental illness connection: the science of dual diagnosis

**Fig. 3:**
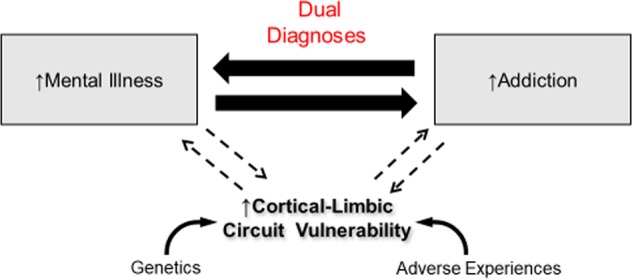
The addiction–mental illness connection. Mental illness and addiction are highly comorbid diseases that have been demonstrated to be deeply interconnected through alterations in shared neural circuity and neurobiology, genetic risks, and environmental-developmental risk factors. The biological-causal relationship is also bidirectional: having either mental illness or addiction involuntarily/biologically increases the likelihood and severity of acquiring the other.

Addictions and mental illnesses are tightly inter-connected diseases both within individual brains^67–70^ and on population levels^71–74^ (Fig. 3). The biological causal connection is involuntary and general across many types of addictions and mental illnesses. This causal connection is also bidirectional: having either illness category increases the risk of acquiring the other, and having either also worsens the severity of the other^67,75^. This bi-directionality of causality is sufficient to explain both the dense epidemiological overlap between these illnesses and the fact that dual diagnosis patients face greater degrees of virtually all quantifiable illness consequences compared to their single disease counterparts, including poorer psychosocial functioning, additional medical comorbidities and service utilization, increased risk for homelessness, suicide, overdose, and premature death in general^40,76,77^.

The involuntary causal nature of the mental illness–addiction connection is born out in both animal and human research. Animal models of mental illness show abnormal short and long-term motor and motivational reactions to addictive drug exposures whether they are self-administered or delivered by researchers^78,79^. In humans, the risk of acquiring addiction, illness severity, and resistance to treatment is not related to an individual’s choice or reasons for initially trying a drug, or their capacity to express a desire to quit using; rather, addiction risk and illness burden is increased with greater mental illness severity^76,80,81^.

An important clue about the mental illness–addiction connection is that this relationship is not specific to any one addictive drug type, or intoxicating profile. For example, it is constructive to compare nicotine and opioids, which have very different psychoactive properties, but are nevertheless both highly addictive, and very deadly as addictions. Half of all cigarettes smoked in the U.S. are consumed by only about 15% of the population—those with some form of mental illness^82^. Likewise, about half of all prescribed opioids in the U.S. are used by a similar fraction of the total population—those with mental illness^83^. Even prior to the modern opioid epidemic, large population-sample studies spanning decades have consistently replicated the observation that substance disorders are elevated 2–8-fold above the general population across all of the major mental illnesses including psychotic disorders, bipolar disorder, major depression, dysthymia, and personality disorders^72,74,82,84–86^. And again, this connection, within each of these diagnostic categories, is not drug specific. Rather it is general to addiction risk that spans many drug types. Thus addictions involving nicotine, opioids, alcohol, stimulants, and cannabinoids are all elevated across a wide range of different forms of mental illness^72,73,87–90^. As a reflection of this phenomena, multiple addictions also routinely occur together with mental illnesses in both individuals and across whole clinical populations^91^. These ‘high-order’ dual diagnosis illnesses have been replicated in animal modeling, reflecting the way mental illness biologically increases addiction risk simultaneously across different addictive drugs even with differing intoxication profiles^70^.

Only recently has the biological connection between mental illness and addiction been recognized or understood as a bidirectional causal problem. While it has been known for decades that alcohol and drug use can induce psychiatric symptoms in otherwise healthy people, or worsen mental illness, it has been less clear if and how the opposite happens, that is, how mental illness itself alters the progression of addiction disease. However, animal modeling and human neuroimaging evidence accumulating since the 1990s has made it clear that mental illness and addiction are inextricably linked on anatomical and pathophysiological levels. Abnormal neurocircuitry present in mental illness, even before drug exposure, changes thresholds for becoming addicted and increases the speed of disease progression and illness severity^67,76,79,92^. This reality, being quite general to many combinations of addictions and mental illness, likely reflects a fundamental design motif (and vulnerability) of the mammalian brain. All of the major limbic neural networks, including the prefrontal cortex, hippocampal formation and amygdala—which are each implicated across the major forms of mental illness—densely project via glutamatergic connections into the key motivational center of the brain, where addiction disease is primarily centered—the nucleus accumbens (or ventral striatum)^67,93^. This design allows all of the higher order neural networks of the brain (that represent and adapt contextual memory, emotion, social-attachment, and decision-making functions) to converge in the nucleus accumbens, where they contribute to the neural representations and computations that generate motivation^92^. Such an architecture optimizes the brain’s capacity to integrate past experiences and current cognitive states to best prioritize, sequence, select, and adapt motivated behavior for long-term success and survival. But, when one or more of these distributed limbic input territories to the nucleus accumbens are structurally and functionally impaired—as in mental illness—the function of the nucleus accumbens itself is altered. This causes the acute and long-term neuroplastic effects of dopamine neurotransmission that is pathologically evoked by addictive drug use, to have a much more profound, long lasting, and devastating effect on motivated behavior^68,79,94,95^.

Beyond this core frontal-cortical/temporal-limbic/ventral-striatal circuity, additional cortical and deep brain regions are increasingly implicated as neuroanatomical zones where addiction and mental illness pathologies are directly tied into one another. For example, recent studies have implicated the lateral septum^96^, the habenula^97^, and the insula^98^ in functions that integrate cognitive, emotional, motivational, and appetitive control systems. Thus across a broad swath of primary cortical-striatal and accessory limbic networks of the brain, mental illness, and addiction vulnerability are wired into one another and are mechanistically unified to such an extent that in many cases it is biophysically impossible to have one without increasing vulnerability to the other. Hence addiction vulnerability is a biologically intrinsic symptom of mental illness that pervades different types of psychiatric diagnoses.

Consistent with this integrated neurocircuit understanding of dual diagnosis, we are now gaining more insight into how both genetic and environmental/developmental risk elements contribute to this core neurocircuit vulnerability that gives rise to both mental illness and addictions^69,99–102^. For example, twin studies and large genome-wide association studies have identified shared genetic risks for alcohol addiction and a range of mental illnesses that are frequently comorbid with addictions including schizophrenia, attention-deficit hyperactive disorder, and major depression^103,104^. Similarly, childhood neglect, abuse and trauma, which collectively represent the major non-genetic (i.e. environmental) root cause of adult psychopathology, are also robust risk factors for acquiring addiction^71,105–107^. With this evidence, it is becoming clear that adverse childhood experiences and genetic determinants work in concert during pre-adult neurodevelopment, within the same cortical-striatal-limbic brain systems, to produce both mental illness and addiction vulnerability^108^.

## The science of dual diagnosis: a failure in translation

Although mental illness and addiction share common etiological factors, have overlapping neuroanatomies and neurodevelopmental trajectories, show bidirectional causality and a tight epidemiological overlap (such that dual diagnosis patients represent the majority of all behavioral health patients) these patients are often referred to as ‘system misfits’ because of a lack of access to integrative treatment services^40,109,110^. The highly fragmented behavioral healthcare system has been built and operates in contradiction to this science: Federally sponsored psychiatric research (e.g. NIDA and NIAAA vs. NIMH), professional training, treatment teams and infrastructures, cultures of care, and insurance reimbursement remain largely divided by a line that separates the addiction vs. mental health-treatment fields^108,111^. Only 18% of addiction treatment programs and 9% of mental health-treatment programs are considered dual diagnosis capable^111^, while many cultural and structural barriers to mental health–addiction integration persist despite decades of effort to create a national system of integrated dual diagnosis treatment^109–112^.

The failure to translate the science of dual diagnosis into driving the integration of clinical training and treatment delivery—so that mental illnesses, addictions, and their bidirectional causal dynamics can be diagnosed and treated by one healthcare team as comprehensively and efficiently as possible^108,112^ has had significant public health and social consequences. The iatrogenic opioid epidemic has revealed the consequences of low medical professional awareness of addiction, and disregard for the risk for addiction in persons with *mental illness*, contributing to the over prescription of high dose/chronic opioids specifically to people with mental illness and addictive disorders^113,114^. This targeting of vulnerable brains with opioid overprescribing, termed by Sullivan et al. as “Adverse Selection”, has occurred alongside declines in behavioral health expertise, workforce, and treatment access and an intensification of the War on Drugs^115^.

## Dual diagnosis and acceleration of the penrose effect by the war on drugs

**Fig. 4:**
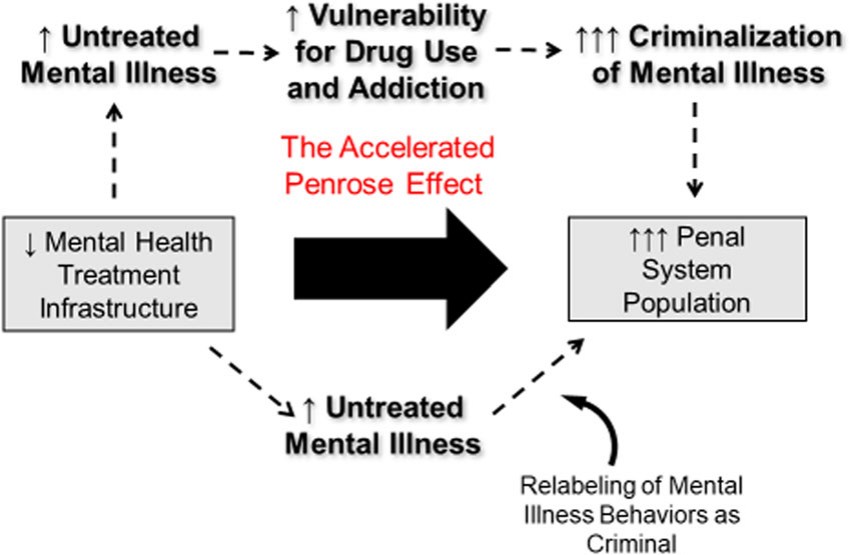
Acceleration of the Penrose Effect by the War on Drugs. Through the fundamental neurobiological and clinical connections between addiction and mental illness, the criminalization of individuals with addiction promoted by the War on Drugs leads to an acceleration of the Penrose Effect. This ultimately hastens the decline and fragmentation of behavioral healthcare, while producing explosive growth of the U.S. penal system, even as neuroscience that is advancing our understanding addictions and mental illnesses as brain diseases, remains increasingly disconnected and unapplied to behavioral health care.

As reviewed thus far, mental illness and addiction are brain diseases that, via the Penrose Effect and the War on Drugs, have been marginalized by the healthcare system, but absorbed by the legal system as reflecting criminal behavior^21,22,58,61,66^. At the same time, however, the neuroscience of dual diagnosis has characterized mental illness and addiction as bi-directionally causative and tightly interlinked brain diseases^68,79,99,106,108^. A key implication of this neuroscience is that the Penrose Effect and the War on Drugs are not merely parallel social processes, but are interlinked and mutually reinforcing. With a decline of mental health-treatment infrastructure, we expect more people incarcerated for behaviors resulting from untreated mental illness. Simultaneously, because these populations are also biologically, involuntarily predisposed to acquiring addictions, then increasing numbers of people with mental illness living without access to good mental health services likely increases the numbers of patients who are acquiring addictions, and demonstrating behavioral consequences of untreated addiction, that are also criminalized by the War on Drugs. Then, as the Penrose Effect is accelerated by the War on Drugs, there is an ever greater transfer of resources, workforce development, public funds, and infrastructure away from treating either illness class as healthcare issues, and instead, toward enforcement, prosecution, conviction, and mass incarceration of untreated patients. These trends would thus intertwine to create a feed-forward amplification of untreated addictions and mental illness to crisis proportions, even to the extent where advances in the neuroscience of these disorders remain largely untranslated by an increasingly deficient and fragmented behavioral healthcare system (Fig. 4).

Recent studies suggest a synergy between the Penrose Effect and the War on Drugs has occurred in the U.S. and internationally, in which mentally ill people are not merely transferring out of treatment beds and into incarceration beds in 1:1 ratios. To the extent that mentally ill people, due to their addiction comorbidities, have been intensively subject to harsh drug penalties and the criminalization of addiction, we might expect a much more disproportionate exchange between treatment and penal capacities. Thus, in a multinational South American study examining the Penrose Effect from 1990 to 2012, for every one mental health treatment bed that disappeared, five more individuals ended up in prison, where, like in the U.S., very high prevalence rates of mental illness and addiction disorders have been identified^20^.

A national U.S. survey showed that individuals with a dual diagnosis were nearly 7.5 times more likely to be arrested in the last 12 months, compared to healthy individuals, with only 1.8 or 5.3 fold increases in arrests in persons with only a mental illness or an addiction^116^. In a Seattle study, substance use seems to directly mediate the relationship between a recent reduction in psychiatric beds and an increase in jail detention for those with mental illness^32^. Accordingly, a lack of substance disorder treatment throughout mental healthcare is an important risk factor for incarceration of patients with dual diagnosis^77^. Among individuals with a mental illness, co-occurring substance use increases behaviors that are criminalized (purchasing, dealing) leading to arrests and incarcerations to such an extent that having a dual diagnosis has become a normalized characteristic of prison populations^117,118^.

These observations suggest that deinstitutionalization and the War on Drugs may have intersected to accelerate the Penrose Effect, though multiple dynamics, at the core of which is a fundamental epidemiological and neurobiological linkage between mental illness and addiction. While Penrose may have had keen insight into the inverse relationship between psychiatric beds and prison populations 80 years ago, he was unaware both of the neurobiology of drug addiction and its biological link with mental illness, which have only been described in the last 30 years. Thus, he could not have predicted the impact the War on Drugs might later have, to not only accelerate the incarceration of mentally ill people (via their comorbid association with addiction), but the degree to which the resulting growth of the penal system might actually interdict the development and deployment of science-based treatments, and contribute adversely to the degradation of the behavioral healthcare system itself^60,119^.

## The war and drugs as harm amplification

**Fig. 5:**
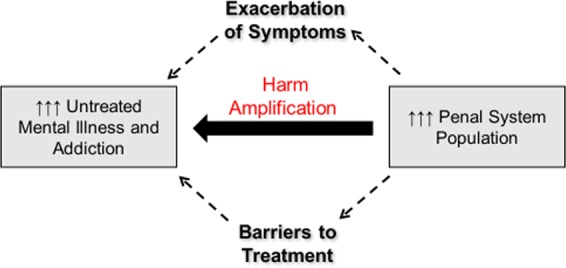
Harm amplification. The mass criminalization of addiction and mental illness has created a large population of individuals with these diseases in a massively overgrown penal system. This enlargement represents a barrier to treatment access for mental illness and addiction for both un-incarcerated and incarcerated patients alike, through reinforcing stigma and financial diversion away from treatment into criminalization. For imprisoned patients, the penal system may exacerbate symptomology and harm treatment access in either disease domain via isolation/confinement practices, victimization by other prisoners, disrupting family attachments, hampering reentry into the community, and impairing access to health insurance, employment and housing from months and years after incarceration.

Aside from the channeling of public funds (and public regard) away from behavioral healthcare, into criminal justice approaches for addressing behaviors resulting from untreated mental illness and addictions^60^, there is evidence that the War on Drugs may directly worsen mental illness via a process that can be termed as ‘Harm Amplification’ (Fig. 5). Many treatments in mainstream medical care, particularly for conditions that are difficult to cure quickly or directly, pursue ‘Harm-Reduction’ strategies, where the therapeutic intervention seeks to limit the negative overall functional impact and/or spread of the injury or disease to other body organs. A cast on a broken arm, insulin for diabetes, and opioid maintenance treatments for opioid addiction are all evidence-based and highly effective Harm-Reduction interventions. Oppositely, the core strategy of the War on Drugs, through punishing drug use and closely related behavior, represents Harm Amplification, where various primary and secondary damages associated with having the disease are deliberately compounded by the criminal justice system (e.g. via public humiliation, financial penalties, and incarceration), in hopes that this will motivate effort in the individual to abandon their addiction.

Beyond the removal of mentally ill/addicted patients from access to mental health and addiction care more generally available at higher standards outside of jails and prisons^39,58,60,120–122^, incarceration settings frequently tend to rely on behavioral control interventions that encompass isolation techniques^60^, which are well known to exacerbate brain abnormalities and behaviors that are present in mental illness and addiction^79,123^. Additionally, mentally ill patients face greater psychological and physical victimization in prison and disproportionate rates of solitary confinement, symptom worsening, and higher rates of suicide in prison^39,60,120,124,125^.

Dual diagnosis patients may be particularly adversely affected by incarceration in terms of long-term outcomes^126,127^. While lack of adequate addiction treatment is a major risk factor for re-arrest following release^58,60^ individuals with dual diagnosis post-incarceration have a higher risk for significant injuries (including drug-related and self-harm) compared to their single disease counterparts^77,126,128^. Finally, the long-term and ‘collateral consequences’ of criminalization of mental illness and addiction are significant. Life post-incarceration often includes problems with reestablishing family bonds, extended unemployment, lack of housing and access to health insurance, all of which are essential for patients (and a well-functioning behavioral health system needed to serve them)^129,130^. In the absence of a strong outpatient system and physician workforce that can provide integrated/longitudinal mental health and addiction care, a large portion of individuals with mental illness and addiction are stuck in repeated cycles between emergency rooms and correctional facilities without adequate care^39,58,60,77,120,124^. In turn, these patients and their brain diseases are viewed, even by the healthcare system and the insurance industry, as recidivistic, undeserving of healthcare resources, and essentially criminal. These trends have contributed to a healthcare system that has been negligent of addiction as a disease that is pathophysiologically connected with mental illness. The seeds of the modern iatrogenic opioid epidemic were thus planted, leading to unprecedented levels of opioid addiction, mortality, and criminal-legal consequences for addictions, especially in mentally ill people^63,114,131^.

## Conclusion

Eighty years ago, Penrose warned that reducing mental health treatment infrastructure would result in increased prison populations. Dr. Penrose died just prior to the advent of the War on Drugs which has likely functioned as an unexpected accelerant of the Penrose Effect in the modern era. Underpinning this acceleration, there exits fundamental neurobiological, and epidemiological connections between addictions and mental illness, as characterized by a broad base of scientific evidence that is still not being adequately translated to inform integrated behavioral healthcare training, service delivery, and treatment research. The associated decline and fragmentation of behavioral healthcare, occurring despite major advances in our neuroscientific understanding of addictions and mental illness as brain diseases, is not only a public health calamity, but jeopardizes the future of psychiatric neuroscience itself, as this science continues to show that it cannot be effectively translated to clinical care, or, if there is no longer a functional behavioral health system to translate to.

While having focused on the Penrose Effect as being accelerated by the War on Drugs, it is also likely that the reverse is simultaneously true: The War on Drugs and associated consequences are probably also amplified by the Penrose Effect. Increased incarceration of those with untreated addiction would be exacerbated by a lack of treatment resources for mental illness, and the even greater paucity of integrated treatment services of addictions in mentally ill people. In this way, a bidirectional amplification of the Penrose Effect and the War on Drugs, happening on the sociological-population scale parallels the bidirectional causal connection and amplification that exists between mental illness and addictions within the brains of millions of individuals. The synergistic criminalization of addiction and mental illness, coupled with a failure in translating the neuroscience of dual diagnosis to building a strong national system of integrated dual diagnosis care, represents a critical failure of the U.S. healthcare system. Large scale signs of this failure continue to emerge both in terms of the iatrogenic opioid epidemic, and the recent decline in the life expectancy for all Americans, which is the first such decline in the era of modern medicine. This decline is remarkable not only for being unique among advanced nations, but also for principally reflecting increased mortality due to untreated addictions and mental illness^132–135^.

From this big picture perspective, we can also infer that mass incarceration and the modern healthcare crisis may also be interlinked and even mutually reinforcing, as mediated by the Penrose-War on Drugs Effect. Given the depth and scope of expensive-to-treat injuries and chronic medical diseases of the body that the U.S. healthcare system is incentivized to treat (that frequently result from untreated addictions), the American people pay for a healthcare system that performs about as well as Cuba’s, but at 14 times the cost^136^. Yet, ultimate responsibility for solving this health crisis, must fall on the healthcare system itself and not the criminal justice system, which is equipped with professionals trained to carry out justice, enforce laws, and punish crime, *not to provide healthcare*^21,23^.

This big picture perspective may provide guidance for a new national strategy needed to restore the cost-effectiveness of U.S. healthcare and to decisively end mass-incarceration. Reversing the Penrose Effect, ending the War on Drugs (as a criminal-legal strategy), and treating addictions and mental illness before they transform into extremely expensive, lethal injuries, multi-organ diseases, and/or prosecutable behavior, will require a non-superficial rebuilding of the behavioral health workforce and infrastructure. The neuroscience of dual diagnosis should be expanded and advanced as a major (rather than largely excluded) NIH research domain as contributed to from across the NIAAA, NIDA, and NIMH portfolios. This would better align their research missions with addressing mental illness-addictions comorbidity as the mainstream public health crisis that it actually represents, allowing greater progress in the development of new integrative and more parsimoniously effective preventative and treatment measures for dual diagnosis conditions. Moreover, the science of dual diagnosis available to us already, which so clearly highlights the fallacy of segregating mental health from addiction expertise and services, needs to be practically and fully translated into a new system of clinics (and closely linked inpatient units) that fully integrate the diagnosis and treatment of mental illnesses and addictions, as provided by cross-trained physicians, nurses, and therapists^108,112^. This should occur along with removing the responsibility placed on the criminal justice system for paying for, dictating standards of care for, and providing addiction and mental health treatment. If occurring in parallel with efforts to achieve full parity of insurance coverage for behavioral health on par with medical care, in a way that completely separates insurance coverage and standards of care from criminal-legal history, or incarceration status, the U.S. might truly arrive at having the most advanced and cost effective healthcare system in the world.
